# Maximizing Fiber Utilization for Sustainable, Efficient Ruminant Production

**DOI:** 10.3390/ani16091340

**Published:** 2026-04-27

**Authors:** Zhiyuan Ma, Ting Liu, Weiwei Wang

**Affiliations:** 1 College of Pastoral Agriculture Science and Technology, Lanzhou University, Lanzhou 730020, China; 2 College of Animal Science and Technology, Gansu Agricultural University, Lanzhou 730070, China; liuting@gsau.edu.cn; 3 College of Animal Science, Guizhou University, Guiyang 550025, China; wwwang3@gzu.edu.cn

## 1. Introduction

The dual pressures of a growing global population and the urgent need to mitigate climate change necessitate a fundamental shift in livestock production. For ruminants—a critical source of meat and milk—this shift hinges on their unique ability to convert fibrous, inedible plant materials into human food. However, the inherent inefficiency in fiber digestibility within the ruminant digestive system represents a major bottleneck [1]. Therefore, further research on ruminant fiber utilization represents not merely an incremental improvement but a cornerstone for sustainable intensification. Maximizing the ruminant’s natural foregut fermentation process directly enhances feed efficiency [2], reduces the environmental footprint [3], and improves the economic resilience of production systems by transforming waste streams into nutrients.

We are pleased to present this Special Issue, which comprises twelve insightful research articles and reviews addressing this core challenge. This collection reflects the vibrant, multidisciplinary efforts that are being made to unravel the complex interactions between feed processing, rumen function, microbial ecology, and animal performance.

## 2. An Overview of Published Papers

### 2.1. Enhancing Fiber Degradation Through Rumen Microbiome Manipulation and Feed Processing

This theme focuses on directly improving the accessibility and fermentability of fibrous substrates. Research demonstrates that the physical and chemical structure of fiber is a primary determinant of its utilization. Mixing ensiled rape straw with whole crop corn can effectively disrupt its lignocellulose matrices, significantly enhancing their ruminal degradability [Pu, et al., Contribution 8]. Beyond physical processing, targeted nutritional modulation of the rumen ecosystem is key. Another contribution [Xu et al., Contribution 4] reveals that yeast culture supplementation in pelleted diets promotes a more favorable rumen environment for fibrolytic microbes, aiding rumen development and metabolic function. These results are consistent with reports from other researchers that yeast cultures help stabilize ruminal pH [1], since excessively low pH is a major factor that impairs fibrolysis in the rumen [4]. Furthermore, foundational work [Lv et al., Contribution 7] systematically reviews the rumen as a reservoir of specialized lignocellulolytic microorganisms, providing a crucial resource for future bioaugmentation strategies. Importantly, research on adapted species like Tibetan sheep [Zheng et al., Contribution 11] highlights how the rumen microbiome dynamically responds to dietary shifts, offering insights into host–microbiome co-evolution. A study on dairy cows [Xia et al., Contribution 9] establishes a direct link between specific rumen microbiota features and host feed efficiency, underscoring the microbiome’s functional role in fiber energy harvest. Interestingly, nutritional strategies traditionally believed to inhibit the proliferation of fibrolytic bacteria in the rumen, such as increasing concentrate supplementation [5], can also promote ruminal fiber degradation when added at appropriate levels. Concentrate supplementation can increase the abundance of fiber-degrading bacteria and enhance the fiber-degrading capacity in the rumen of lactating Tibetan sheep during the cold season [Yang et al., Contribution 10]. Fats, especially those rich in polyunsaturated fatty acids, are generally regarded as a major nutrient that inhibits fibrolytic bacteria [6]. However, supplementing hempseed oil (up to 200 g/d) in the diet of lactating buffaloes slightly reduced their dry matter intake, but maintained the stability of rumen fermentation and specifically altered the abundance of *Acetobacter* associated with acetate production [Gu et al., Contribution 2]. This indicates that the hempseed oil exerted no negative effects on the rumen environment required for fiber degradation. Collectively, these results suggest that nutritional balance in the rumen is crucial for maintaining fibrolytic function.

### 2.2. Optimizing Dietary Formulation for Rumen Function, Health, and Productivity

The papers with this theme aim to determine how to balance dietary components to sustain optimal rumen conditions for fiber digestion while supporting animal performance. A central tenet is maintaining rumen health and stability. Research on sheep [Zhang et al., Contribution 1] precisely defines the optimal balance between forage neutral detergent fiber and rumen-degradable starch, providing a dietary formulation guideline to prevent acidosis and support continuous fermentation. The inclusion of high-quality forages remains vital, as shown in [Xia et al., Contribution 9], where partial replacement of alfalfa hay with silage improves dairy cow performance, demonstrating the value of forage quality and preservation. Concurrently, addressing anti-nutritional factors in alternative protein sources is essential for overall diet quality; a comprehensive review [Yan et al., Contribution 5] details strategies to mitigate these factors in plant protein feeds, ensuring a balanced nutrient supply. This holistic approach to diet formulation directly supports sustainable intensification by maximizing nutrient extraction from the complete diet.

### 2.3. Mitigating Environmental Impact Through Integrated Nutritional Strategies

A core objective of modern ruminant production is to reduce its environmental footprint, particularly methane emissions, without compromising productivity. The research presented offers several promising pathways for achieving this. This bibliometric analysis by Zheng et al. [Contribution 3] identifies that research on rumen microbiota and methane mitigation has evolved from traditional animal nutrition studies focused on phenotypic indicators like digestibility to a deeper focus on microbial mechanisms (e.g., protozoa and methanogens), with enteric methane recognized as being primarily produced via the anaerobic fermentation of ingested carbohydrates, including fiber, in the rumen. One study [Li et al., Contribution 12] investigates nitrate supplementation as a hydrogen sink, directly competing with methanogenesis and demonstrating significant methane reduction potential. Similarly, work on yeast culture [Yi et al., Contribution 6] confirms its role in modifying fermentation patterns, leading to lower methane production. Crucially, these mitigation strategies are examined in the context of overall system efficiency. The aforementioned study on dairy cow feed efficiency [Xia et al., Contribution 9] explicitly connects dietary fiber–starch ratios with both methane yield and milk output, illustrating the possibility of synergistic gains. This integrated perspective is essential for developing practical, adoption-ready solutions that align environmental stewardship with economic viability for producers.

## 3. Conclusions

Bringing together twelve insightful research articles and reviews, this collection represents a multidisciplinary effort to unravel the complex interactions between feed processing, rumen function, microbial ecology, and animal performance. This work aims to enhance the efficiency of converting fibrous, inedible plant materials into human food. The published papers are organized around three key themes, collectively offering valuable insights for optimizing ruminant production systems. The first theme focuses on enhancing fiber degradation through rumen microbiome manipulation and feed processing, demonstrating that physical processing (e.g., mixed ensiling of rape straw and whole crop corn) can disrupt lignocellulose matrices, while targeted nutritional modulation (e.g., yeast culture supplementation, appropriate concentrate addition, and hempseed oil supplementation) can create a favorable rumen environment for fibrolytic microbes without compromising fiber degradation. Research on adapted species like Tibetan sheep and dairy cows further highlights the dynamic response of the rumen microbiome to dietary shifts and its functional role in fiber energy harvest. The second theme is the optimization of dietary formulation, emphasizing the importance of balancing forage-neutral detergent fiber and rumen-degradable starch to maintain rumen health, as well as the value of high-quality forages and strategies to mitigate anti-nutritional factors in plant protein feeds, ensuring balanced nutrient supply and supporting animal performance. The third theme focuses on mitigating the environmental impact through integrated nutritional strategies, identifying the shift in methane mitigation research from phenotypic indicators to microbial mechanisms and presenting promising solutions such as nitrate supplementation and yeast culture to reduce enteric methane emissions while maintaining system efficiency. Collectively, the findings in this Special Issue emphasize that nutritional balance in the rumen is crucial for maintaining fibrolytic function. Integrated approaches combining feed processing, microbiome manipulation, and dietary optimization are key to achieving sustainable intensification. These strategies enhance feed efficiency, reduce environmental footprint, and improve the economic resilience of ruminant production systems.
